# Spatiotemporal variation evaluation of water quality in middle and lower Han River, China

**DOI:** 10.1038/s41598-022-16808-w

**Published:** 2022-08-19

**Authors:** Lele Deng, Kebing Chen, Zhangjun Liu, Boyang Wu, Zekun Chen, Shaokun He

**Affiliations:** 1 Jiangxi Academy of Water Science and Engineering, Nanchang, China; 2 State Key Laboratory of Water Resources & Hydropower Engineering, Wuhan University, Wuhan, China; 3 Bureau of Hydrology, Changjiang Water Resources Commission, Wuhan, China; 4 TUM Department of Civil, Geo and Environmental Engineering, Technical University of Munich, Munich, Germany; 5 School of Environmental Science and Engineering, Southern University of Science and Technology, Shenzhen, China

**Keywords:** Environmental impact, Environmental sciences, Environmental social sciences

## Abstract

As the water source for the middle route of the South-to-North Water Transfer Project, the Han River in China plays a role of the world’s largest inter-basin water transfer project. However, this human-interfered area has suffered from over-standard pollution emission and water blooms in recent years, which necessitates urgent awareness at both national and provincial scales. To perform a comprehensive analysis of the water quality condition of this study area, we apply both the water quality index (WQI) and minimal WQI (WQI_min_) methods to investigate the spatiotemporal variation characteristics of water quality. The results show that 8 parameters consisting of permanganate index (PI), chemical oxygen demand (COD), total phosphorus (TP), fluoride (F-), arsenic (As), plumbum (Pb), copper (Cu), and zinc (Zn) have significant discrepancy in spatial scales, and the study basin also has a seasonal variation pattern with the lowest WQI values in summer and autumn. Moreover, compared to the traditional WQI, the WQI_min_ model, with the assistance of stepwise linear regression analysis, could exhibit more accurate explanation with the coefficient of determination (R^2^) and percentage error (PE) values being 0.895 and 5.515%, respectively. The proposed framework is of great importance to improve the spatiotemporal recognition of water quality patterns and further helps develop efficient water management strategies at a reduced cost.

## Introduction

High-quality water resources are of crucial importance to maintain ecological integrity and promote sustainable socio-economic development^1,2^. However, water quality issues have been more intricate than ever before due to different ecosystem pressures from rapid urbanization and population explosion. Water deterioration has also become a public urgent concern worldwide and a serious threat to people’s livelihoods^3,4^. Taking China for illustration, there were 874 records of local water contamination during 2006 ~ 2016, resulting in great troubles for domestic life and making millions of economic loss^5^. Reasonable evaluation of water quality variation has been demonstrated as a practical tool for water quality warning and protection^6–8^.

Indeed, lots of efforts have been made to investigate reliable information of water quality variation^9–11^. The water quality assessment based on hydro-chemical monitoring methods and data sampling underlies the geological heterogeneity of water quality, the understanding of human activities and the control of water contamination^12,13^. There have been considerable research for water quality evaluation in recent years, including the single-factor evaluation method^14^, Namerow pollution method^15^ and water quality index (WQI) method^16,17^. Of them, the recent WQI method takes full advantage of water quality parameter information and converts all parameters into a clear normative status value of water quality. It has been a prevailing approach for water quality assessment in a wide range of studies. The calculation of WQI has also experienced modification and has been developed in different ways. For instance, the National Sanitation Foundation WQI (NSFWQI) that comprises 9 parameters has been applied in different regions across the world^18^. Bascaron WQI^19^ and Canadian WQI^20^ that resemble the NSFWQI also had wide applications in the various background.

However, the downside of inflexible water quality parameters limits the application of aforesaid different WQIs. Some studies are devoted to identifying the key water quality parameters and developing more efficient WQI methods at a low-cost level. The minimum WQI (WQI_min_) model consisting of the key water quality parameters that can deal with the information redundancy and high cost has been demonstrated effective. A highly linear correlation relationship between WQI and WQI_min_ has been documented^21,22^, indicating that WQI_min_ has a strong potential to reflect the variation of water quality economically. However, the results of WQI_min_ models may show substantial differences using various parameters and assigned weights^17,23^. The parameters selection for a WQI_min_ model requires a deliberate response in terms of the specific scientific issue. The stepwise linear regression analysis has been verified robust to identify critical parameters for reduction of data redundancy^14,24,25^, here, it is employed in this study.

As the largest tributary of the Yangtze River in China, the Han River has been subject to water blooms on several occasions. Since the first diatom bloom of the Han River in 1992, there have been 9 water bloom episodes of varying degrees during the period 1998 ~ 2016, all of which concentratedly occurred between February and April. The influenced river reached about 500 km above the estuary in severe cases, and it usually lasted as long as 20 days^26^. Some literatures have reported that water blooms are closely associated with water quality^27,28^. Meanwhile, the implementation of China’s Middle Route of South-to-North Water Transfer Project will enhance the water supply capacity in the north while reducing the downstream flow, which may further exacerbate the water quality deterioration of the downstream environment^29,30^. The unqualified river flow has a direct crash on the drinking water safety in riverine cities such as Wuhan, which has received intensive attention from the local government. Most of the previous studies regarding the middle and lower reaches of the Han River basin typically focused on the optimization of cascade reservoirs operation^31^ or water resources allocation^32^, the water quality has come to stand out in recent years. These studies aimed at analyzing the correlation between water bloom and water quality within a short period, while the temporal and spatial variations in water quality in different times and river sections were less investigated. Furthermore, only a few studies targeting assessing the water quality status but the single factor evaluation method or the Nemerow's pollution index was adopted as the evaluation method^33^. The WQI method had not been applied to Han River, let alone the minimal WQI_min_ method. Consequently, we still lack a thorough and comprehensive understanding of the water quality in this high-profile area. The temporal and spatial variations in water quality, the application of WQI method and the development of the WQI_min_ model consisting of the key water quality parameters need further investigation.

To bridge these gaps, we examined 15 representative water quality parameters collected from 11 monitoring stations during 2015–2017 for investigation of the water quality level. The main goals of this study are (1) to analyze the spatiotemporal variation of each water quality parameter in the study area; (2) to comprehensively evaluate the water quality status as well as the spatial profiles and seasonal patterns using the water quality index method; and (3) to identify the critical parameters and develop a WQI_min_ model for more efficient and cost-effective water quality evaluation.

## Materials and methods

### Study area

Located in the middle of the Yangtze River economical belt, Han River is the largest tributary of the Yangtze River, China. It is usually segmented into three divisions, i.e., upstream from its source to the Danjiangkou Reservoir, middle stream from the Danjiangkou Reservoir to the Jingzhou City, and downstream from Jingzhou to the Wuhan City. The total length of middle and low streams is 676 km, with a drainage area of 64,000 km^2^. Characterized by the subtropical monsoon, the river basin has abundant annual average precipitation from 700 to 1800 mm which concentrates in the summer and autumn seasons.

**Figure 1:**
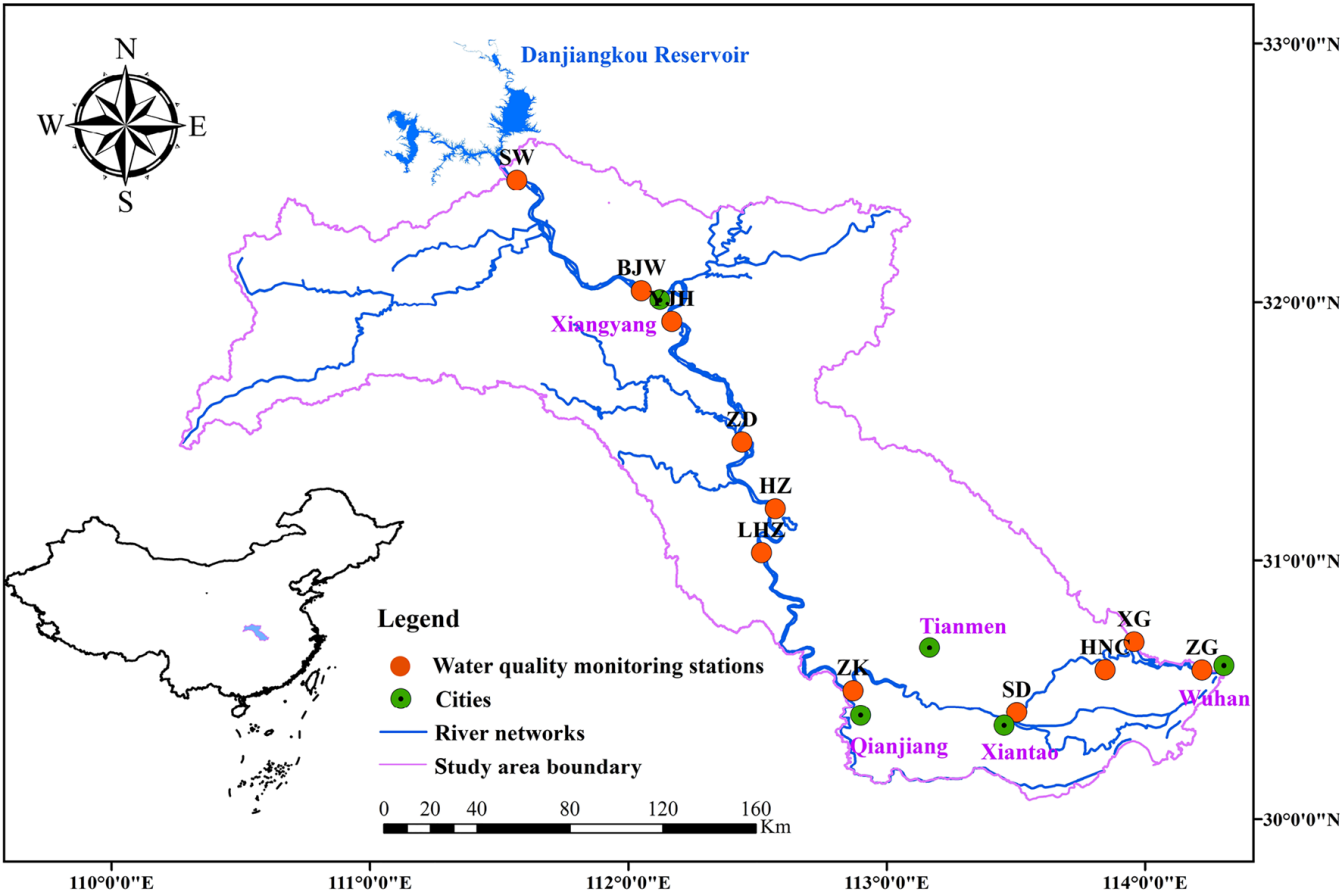
Location of the water monitoring stations in the middle and lower Han River basin. (This figure is generated by ArcGIS10.2 software. URL link: http://www.arcgisonline.cn/).

With huge water resources potential, the area plays a significant role in the provincial granary, industry and national “one belt, one road” construction. Eleven water quality stations are located along the mainstream to monitor the water quality changes. More detailed information is presented in Fig. 1 and further provided in the supplementary material (Table S1).

### Sample measurement and data collection

All archived data in this study were provided by the Hubei Provincial Academy of Eco-environmental Sciences. Water samples were collected on both sunny and cloudy days to eliminate the effect of precipitation. They were sampled monthly from January 2015 to December 2017 in 11 stations, spanning temporal and spatial variation. The *Standard Methods for the Examination of Water and Wastewater*^34^ were used for sample chemistry analyses. There were 15 water quality parameters in total, including pH, dissolved oxygen (DO, mg/L), permanganate index (PI, mg/L), chemical oxygen demand (COD, mg/L), five-day biochemical oxygen demand (BOD_5_, mg/L), ammonia nitrogen (NH_3_^-^N, mg/L), total phosphorus (TP, mg/L), fluoride (F^-^, mg/L), selenium (Se, μg/L), arsenic (As, μg/L), sulfide (mg/L), plumbum (Pb, μg/L), copper (Cu, μg/L), zinc(Zn, μg/L) and mercury (Hg, μg/L). The specific approach for collecting water samples can be found in the standard of the *Guidance on Sampling Techniques*^35^. All the samples were labeled with detailed information using waterproof markers on the bottles to prevent misdiagnosis. Additionally, procedure blank was also used at all the stations to control the accuracy of analyses. With respect to the measurement method, the Hydrolab Datasonde 5 Sensor (USA) was calibrated prior to sampling to measure pH and DO. Except that, titration assembly, UV spectrophotometer (UV 2450), Ion chromatograph system (ICS 2000) and other instruments were also employed for different water quality parameters. More detailed information concerning the instrumental and chemical analysis method could be found on the website of the Ministry of Ecology and Environment of China (http://www.mee.gov.cn/ywgz/fgbz/bz/).

### Water quality index

We used a weighted sum of all fifteen water quality parameters to calculate the WQI, which can be expressed as follows.

1 $$WQI = \frac{{\sum\nolimits_{i = 1}^{n} {(C_{i} P_{i} )} }}{{\sum\nolimits_{i = 1}^{n} {P_{i} } }}$$

where $n$ is the total number of water quality parameters, $C_{i}$ and $P_{i}$ are the normalized value and assigned weight of *i*th parameter, respectively. All weights range between 1 (least impact) and 4 (highest impact on water quality), and the assigned weights listed in Table S2 for relevant water quality parameters have been suggested by previous literature^36–38^. Note that we referred to The *Environmental Quality for Surface Water*^39^ to obtain a normalized value of $C_{i}$ for more accurate evaluation, which was shown as follows:

2 $$C_{i} = \left\{ \begin{gathered} 100 - [\frac{{(T_{i} - S_{i,k} )}}{{(S_{i,k + m} - S_{i,k} )}} \times 20m + I_{i,k} ],\;T_{i} \in [S_{i,k} ,\;S_{i,k + m} ) \hfill \\ 100 - \frac{{T_{i} }}{{S_{i,k + m} }} \times 20m,\;T_{i} \in [0,\;S_{i,k} )\; \hfill \\ \end{gathered} \right.$$

where $T_{i}$ is the measured concentration of *i*th parameter; $S_{i,k}$ and $S_{i,k + n}$ are the standard thresholds of the *i*th parameter at level $k$ and level $(k + n)$, respectively; $I_{i,k}$ is the standard normalization value of the parameter level, i.e., 20, 40, 60, 80 and 100; $m$ is the number of identical values of the threshold, and $m$ is equal to 1 if there is no same threshold. It is worth noting that pH is a special parameter because it has no specific standard threshold, then $C$ value is set to 100 when $6 \le {pH} \le 9$, otherwise it is 0.

WQI value ranges from 0 to 100 and can be classified into five different types as follows: excellent (91–100), good (71–90), medium (51–70), and bad (26–50) and very bad (0–25)^19^. A larger WQI value indicates a better water quality condition. Particularly, the annual period in this study is divided into spring (March to May), summer (June to August), autumn (September to November), and winter seasons (December to the following February). However, the traditional WQI model involves too many parameters and has much uncertainty^17,40^. An improved WQI_min_ model by identifying the key parameters is developed with the benchmark of WQI, and both weighted and non-weighted WQI_min_ models are considered for comparison. The weighted WQI_min_ (WQI_min-w_) model can be calculated by Eq. (1), while the non-weighted WQI_min_ (WQI_min-nw_) model is calculated by Eq. (3).

3 $$WQ{\text{I}}_{\min - nw} = \frac{{\sum\nolimits_{i = 1}^{n} {P_{i} } }}{n}$$

With reference to the data split procedure in previous studies in Nong et al.^24^, Wu et al.^14^ and Uddin et al.^41^, the WQImin models in this study were established using the following steps: The WQI and Ci for each station in 2015 and 2016 were used as “training data” to calibrate the WQI_min_ model while a test period in 2017 was used to verify the model performance.

### Data analysis

The Mann–Kendall (M–K) test has been widely applied to analyze water quality trends in previous studies^24,42^. The calculation processes of the M–K test were shown in detail in Güçlü (2020). The results of the M–K test have illustrated the trends of water quality parameters in this basin and are shown in Fig. S1. The one-way analysis of variance (ANOVA) was applied to verify the spatial differences of parameters. The WQImin models in this study were established using the following steps: (1) The WQI and Ci for each station in 2015 and 2016 were used as “training data” to select the key parameters for the WQImin model; (2) The coefficient of determination (R2) was taken as the goodness-of-fit criterion and the Percentage Error (PE) was used to evaluate the forecasting precision of the WQImin models based on the “testing data” (i.e., the WQI and the Ci for each station in 2017)^44^. The data used in the stepwise multiple linear regression method were pre-processed by the log transformation (i.e., lg(x + 1)).

### Transparency

The authors confirm that the manuscript is an honest, accurate, and transparent account of the study was reported; that no vital features of the study have been omitted; and that any discrepancy from the study as planned have been explained.

## Results

### Water quality characteristics

**Figure 2:**
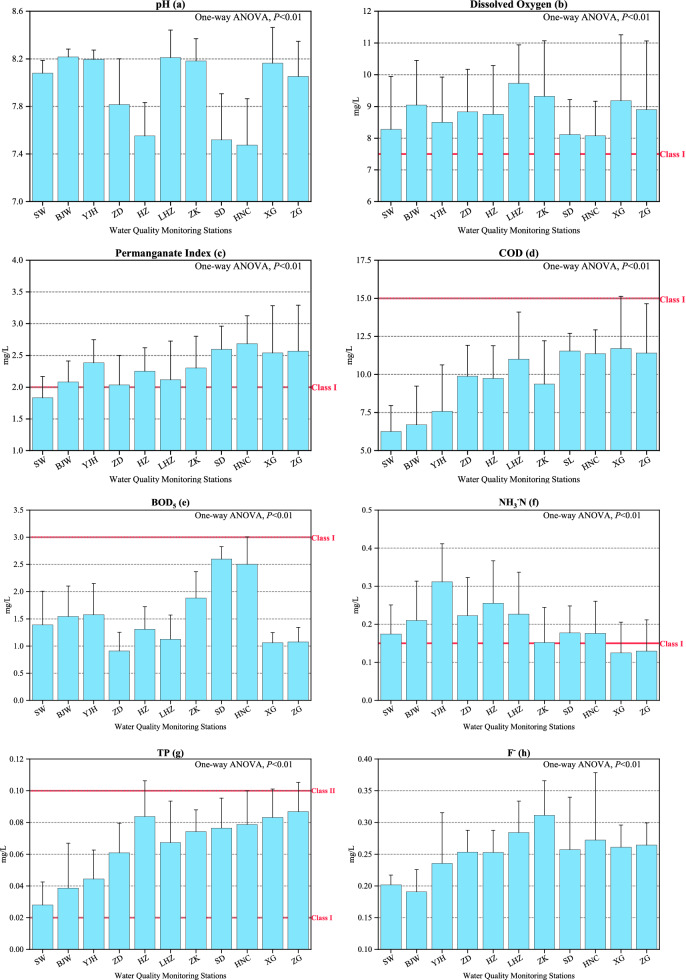

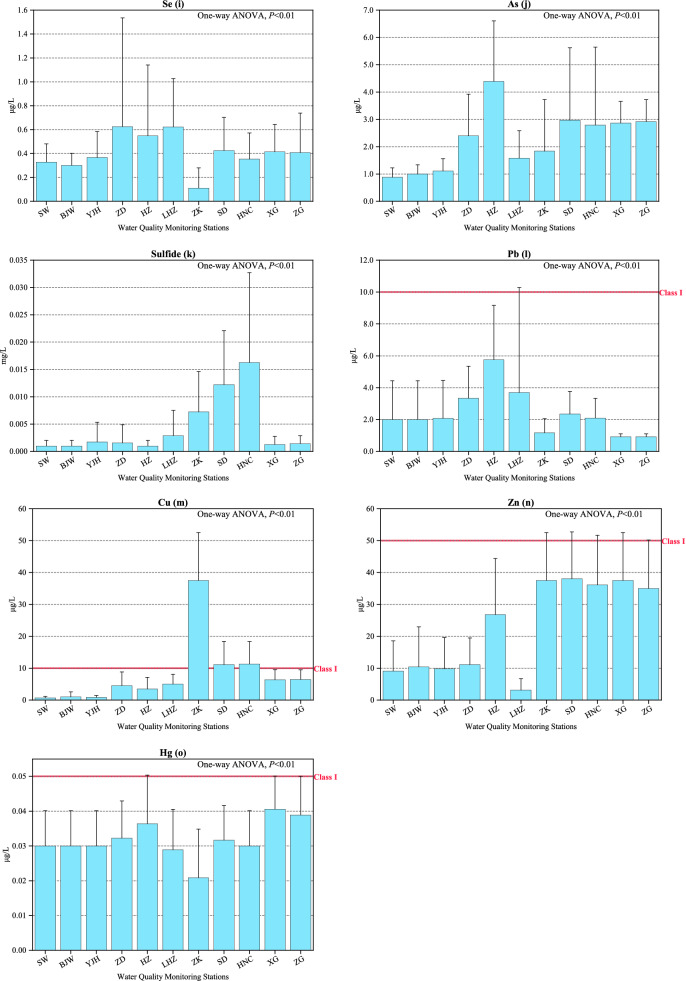
Average and standard deviation of concentration for 15 water quality parameters at each monitoring station during the year 2015 ~ 2017.

Table 1 presents the statistical summary of all water quality parameters in the middle and lower reaches of the Han River basin and Fig. 2 displays their station concentrations.

**Table 1:** Comparison of the variations of the water quality parameters in middle and lower reaches of Han River basin in China from 2015 to 2017 (Avg.: Average; S.D.: Standard deviation).

Parameters	Thresholds of the	2015 (h = 132)			2016 (h = 132)			2017 (h = 132)		
	Class I standards*	Avg. ± S.D	Max	Min	Avg. ± S.D	Max	Min	Avg. ± S.D	Max	Min
pH	N/A	7.86 ± 0.45	8.40	6.70	7.93 ± 0.40	8.80	6.60	8.06 ± 0.29	8.70	6.90
DO (mg/L)	≥ 7.5 mg/L	8.68 ± 1.59	13.40	6.10	8.80 ± 1.75	12.90	5.90	8.91 ± 1.51	12.20	6.10
PI (mg/L)	≤ 2.0 mg/L	2.29 ± 0.51	4.60	1.40	2.28 ± 0.56	4.20	1.30	2.36 ± 0.59	4.40	1.40
COD (mg/L)	≤ 15.0 mg/L	8.97 ± 2.98	21.30	5.00	9.84 ± 3.11	18.00	5.00	10.26 ± 3.25	19.00	5.00
BOD_5_ (mg/L)	≤ 3.0 mg/L	1.55 ± 0.66	2.90	0.50	1.66 ± 0.75	2.90	0.50	1.42 ± 0.67	2.80	0.50
NH3N (mg/L)	≤ 0.15 mg/L	0.212 ± 0.107	0.460	0.020	0.206 ± 0.123	0.600	0.030	0.169 ± 0.077	0.380	0.030
TP (mg/L)	≤ 0.02 mg/L	0.066 ± 0.028	0.150	0.020	0.065 ± 0.025	0.130	0.010	0.066 ± 0.030	0.180	0.010
F- (mg/L)	≤ 1.0 mg/L	0.249 ± 0.056	0.450	0.160	0.263 ± 0.060	0.480	0.130	0.246 ± 0.077	0.570	0.000
Se (μg/L)	≤ 10 μg/L	0.341 ± 0.330	1.000	0.010	0.458 ± 0.590	5.000	0.010	0.427 ± 0.247	2.000	0.020
As (μg/L)	≤ 50 μg/L	2.32 ± 1.44	6.00	0.20	2.60 ± 2.53	17.00	0.02	1.82 ± 1.45	7.00	0.02
sulfide (mg/L)	≤ 0.05 mg/L	0.006 ± 0.007	0.040	0.002	0.003 ± 0.007	0.030	0.000	0.003 ± 0.009	0.090	0
Pb (μg/L)	≤ 10 μg/L	3.16 ± 1.91	5.00	0.50	2.19 ± 2.71	10.00	0.04	1.92 ± 3.91	25.00	0.04
Cu (μg/L)	≤ 10 μg/L	5.82 ± 6.01	20.00	0.20	10.15 ± 12.75	50.00	0.08	8.08 ± 14.01	50.00	0.40
Zn (μg/L)	≤ 50 μg/L	18.85 ± 6.55	60.00	1.00	25.31 ± 21.78	50.00	0.30	25.24 ± 22.74	60.00	0.40
Hg (μg/L)	≤ 0.05 μg/L	0.023 ± 0.007	0.050	0.010	0.038 ± 0.011	0.050	0.010	0.035 ± 0.012	0.050	0.010

*Data from the Environmental Quality Standards for Surface Water^39^. h is the number of the water samples.

#### Biochemical and physicochemical parameters (pH, DO, PI, COD, BOD_5_, and sulfide)

The annual mean pH values were greater than 7.80, and both the maximum and minimum measured pH values occurred in 2016, which were 8.80 and 6.60, respectively. For the annual mean pH, the highest value occurred in BJW station (pH = 8.22), closely followed by LHZ (pH = 8.21). The ZD, HZ, SD and HNC stations obviously observed much lower values than other stations, particularly, the HNC occupied the lowest annual mean pH value of 7.48. The M–K test showed that ZD, HZ, SD and HNC stations with relatively lower pH values had significant upward trends.

The annual mean DO concentrations were higher than the Class I standard (7.5 mg/L). The highest DO concentration of 13.40 mg/L was observed in 2015, while the lowest was in 2016 with the value of 5.9 mg/L. The annual mean DO concentrations increased monotonically from 2015 to 2017. For all stations, LHZ ranked first in terms of the annual mean DO concentration with the value of 9.73 mg/L, while SD and HNC were at the bottom. Surprisingly, XG and ZF observed relatively higher DO concentration compared with the upstream stations. This might be attributed to the influence of the water temperature. Lower river water temperature contributed to the higher DO concentration, vice versa. However, the one-way ANOVA (*p* < 0.01) indicated that there was no spatial difference of DO. The results of the M–K test showed there was only one station, i.e., LHZ, having a downward trend of DO while the remaining (about 91%) exhibited unchanged. Hence, the DO concentration kept relatively stable in all stations during the periods.

PI, COD, and BOD_5_ are all the key parameters for measuring water pollution levels arising from organic compounds. The statistical summary showed the annual mean PI concentration of each year was greater than 2 mg/L, which implied that PI observation couldn’t meet the standard of Class I. The years from 2015 to 2017 observed the maximal PI concentration of 4.6 mg/L, 4.2 mg/L, and 4.4 mg/L, respectively. Figure 2c showed that SW had the lowest mean PI of 1.83 mg/L and the downstream stations had higher PI concentrations than the upstream in general. The last 4 stations all exhibited the mean PI concentration over 2.5 mg/L while the mean concentration of other stations was lower than that value. With respect to COD, the maximum COD of 21.30 mg/L was in 2016 and the minimum was observed the same in each year. The annual mean COD concentration in each year was observed below 11.0 mg/L and less than Class I. Nine consecutive stations showed annual mean COD concentration larger than 7.5 mg/L and the downstream concentration were generally higher than the upstream except ZK station. The M–K test indicated that there were significant upward trends for HNC, XG and ZG stations, which were to be controlled in the following years. In terms of BOD_5_, all the monthly BOD_5_ in all stations were less than 3.0 mg/L and satisfied the threshold value of Class I. The maximum BOD_5_ in each year were almost the same. On spatial scale, SD, HNC and ZK stations showed three highest annual mean BOD_5_ concentrations, respectively, while the lowest occurred in ZD, which implied that SD, HNC and ZK stations exhibited relatively higher organic pollution.

The annual mean sulfide concentrations were far below the threshold of Class I. They were only about 10% of the threshold value (0.05 mg/L). The maximum sulfide occurred in 2017 with a value of 0.09 mg/L. The area had significant spatial differences in sulfide. Four consecutive stations from LHZ to HNC had higher sulfide content than the remaining 7 stations. About 72.7% of stations were analyzed by the M–K test to have significant downward trends for sulfide.

#### Nutrients and soluble ions (NH_3_-N, TP, F^-^)

The annual mean NH_3_-N concentration in each year exceeded the threshold value of Class I-0.15 mg/L. The maximum content was observed in 2016 and the minimum was in 2015, with the values of 0.60 mg/L and 0.02 mg/L, respectively. Figure 2f showed that the consecutive 9 stations exhibited the annual mean NH_3_-N concentration over 0.15 mg/L and only 2 downstream stations met the water quality requirement of Class I. The one-way ANOVA test revealed that no spatial differences were shown from upstream to downstream. But the study area was exposed to heavy pollution caused by TP. The statistical results demonstrated that the TP content of each station was far over the Class I threshold. The maximum TP content was 0.18 mg/L in 2017, followed by 0.15 mg/L in 2015. Figure 2g showed that the area has spatial heterogeneity of TP (one-way ANOVA, *P* < 0.01) and the TP content increased gradually from upstream to downstream. The spatial distribution of TP might be closely correlated with the phosphate industry in Hubei Province and the accumulation of the domestic sewage discharge, the use of fertilizer and pesticide^45^. Furthermore, the TP concentration was closely related to the algal blooms, which should receive much attention.

The soluble ion, F^-^, showed a small variation ranging from 0.246 mg/L to 0.263 mg/L. Compared to TP, the water quality condition for F^-^ was much better. The maximum measured F^-^ concentration in each year from 2015 to 2017 was 0.45 mg/L, 0.48 mg/L and 0.57 mg/L, respectively. Although there was spatial heterogeneity with an increasing trend, all the stations were excellent in terms of F^-^. The M–K test (Fig. S1) indicated that BJW and HNC stations had a significant downward trend.

#### Heavy metal parameters (Se, As, Pb, Cu, Zn and Hg)

The maximum annual mean concentration occurred in 2016 for Se, As, Cu, Zn and Hg with the values of 0.458 μg/L, 2.602 μg/L, 2.096 μg/L, 10.154 μg/L, 25.308 μg/L and 0.038 μg/L, respectively. The remaining Pb parameter had its maximum in 2015. Among the 6 parameters, Se, As and Hg performed best, followed by Pb, Zn and Cu in order. Table 1 showed the maximum value of Pb in 2017, Zn in 2015 and 2016, and Cu in 2015, 2016 and 2017, which were all over the threshold of Class I. The mean concentrations of Se and Hg had some fluctuation yet no obvious spatial changes (one-way ANOVA, *P* < 0.01). The mean concentrations of As increased from upstream to downstream in general except for higher values of ZD and HZ. As for Zn, LHZ had the minimum annual mean concentration and its downstream had higher concentration than upstream. The M–K test showed that there were 6, 2, 2, 6, 5, and 8 stations showing upward trends for Se, As, Pb, Cu, Zn and Hg, respectively.

### Water quality assessment using the WQI method

**Figure 3:**
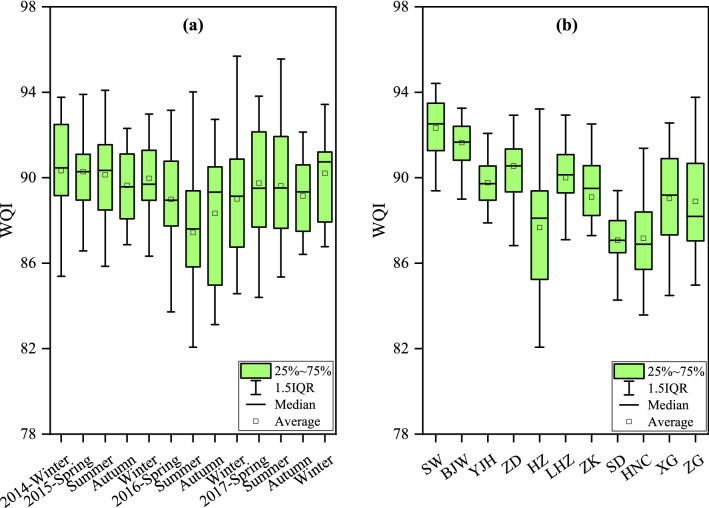
Seasonal (**a**) and spatial (**b**) variations of the water quality index (WQI) in the middle and lower reaches of the Han River basin in China from 2015 to 2017.

The seasonal and spatial patterns of the water quality variations were presented in Fig. 3. In general, the water quality in our study area can be classified into “good” or “excellent” status in most cases, with all average WQI values greater than 87. Figure 3a displayed the seasonal variation of WQI during the monitoring period. The 4 highest seasonal average WQIs occurred in the winter of 2014, the spring and summer of 2015, and the winter of 2017, with values of 90.33, 90.28, 90.13, and 90.21, respectively. The lowest seasonal WQI was in the summer of 2016, indicating the worst water quality condition compared to other seasons. However, it could still be categorized to be “good” water quality status. The seasonal variation of the WQI in 2016 behaved more dramatically than that in 2015 and 2017. In 2015 and 2017, both the seasonal WQI values decreased from spring to summer, followed by a drop to the bottom in autumn and then rose again. In 2016, the lowest WQI values occurred in the summer. The WQI values descended from spring to summer to reach the lowest and then rose to 88.33 in autumn and continued to rise in winter. The lowest WQI in 2016 summer could be tightly associated with the flood during that period^46^. The persistent heavy rain caused severe floods in Han River basin and masses of non-point pollutants entered the channel and contaminated the river. Therefore, a lower WQI occurred in that time.

The spatial profile was displayed in Fig. 3b. The maximum average WQI was in SW and the minimum was in SD and HNC. WQI had certain spatial differences and the upstream stations occupied higher WQI than downstream. It can be attributed to the contaminant accumulation from domesticity, production, and other sources. In addition, YJH owed the most stable water quality condition yet HZ and ZG experienced larger fluctuations.

### The training and test performance of WQI_min_ models

**Figure 4:**
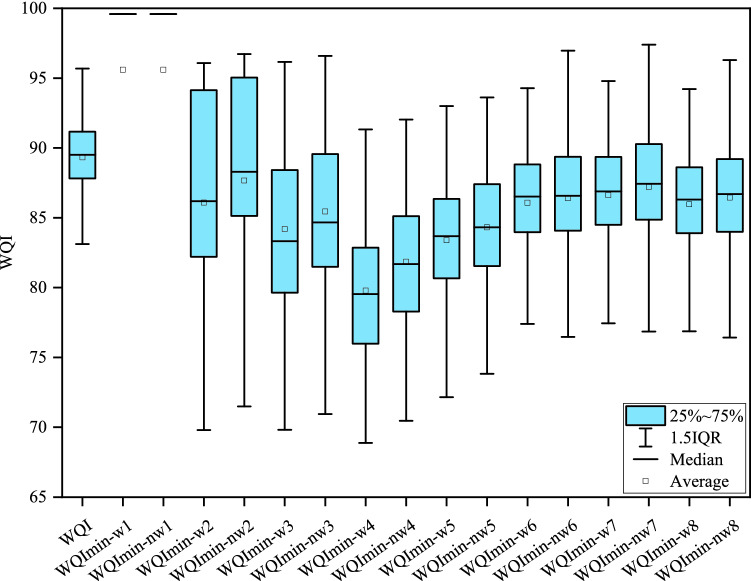
Comparison of WQI and WQI_min_ values based on the training dataset (the parameters for each WQI_min_ model are shown in Table 2).

The results of the stepwise multiple linear regression were shown in Table S3. It showed Zn contributed most to WQI according to the training performance, i.e., Model 1, R^2^ = 0.411, *P* < 0.001; PI, NH_3_^-^N, TP, DO were recognized in sequence and the R^2^ values increased monotonically until up to 0.857. For models with more parameters, such as models 6–8, R^2^ values further improved. To comprehensively evaluate the performance of the WQI_min_ models for assessing water quality, all the models in Table S3 were considered for further investigation. R^2^ and PE were the two selected evaluation criteria for the model performance evaluation. The results indicated that except model 3, other R^2^ values improved with the increasing number of parameters. Compared model 2 with model 3, an extra NH_3_^-^N would decrease R^2^ values but increase PE. Table 2 also showed that the WQI_min-nw_ models had higher R^2^ values and lower PE values than the WQI_min-w_ models, indicating that the former could better explain the WQI variations. Moreover, significant differences between WQI and WQI_min_ existed in the training period as shown in Fig. 4, indicating that the first models were not suitable for WQI simulation, while the remaining models exposed their potential. The average and median of WQI_min-nw7_ model were close to that of the WQI model, which accounted for 90% of the WQI variation with the lowest PE of 2.64%. It was identified as the most suitable WQI_min_ model in this study.

**Table 2:** The parameter selection results of the WQI_min_ models from the stepwise multiple linear regression based on the training dataset (n = 264).

Parameter selection	WQI_min_-_w_ (weighted)				WQI_min_-_nw_ (non-weighted)			
	Models	R^2^	PE(%)	*P*	Models	R^2^	PE(%)	*P*
Zn	w1	0.408	9.909	< 0.001	nw1	0.408	9.909	< 0.001
Zn, PI	w2	0.534	5.595	< 0.001	nw2	0.573	4.395	< 0.001
Zn, PI, NH3N	w3	0.469	6.531	< 0.001	nw3	0.558	5.278	< 0.001
Zn, PI, NH3N, TP	w4	0.597	10.759	< 0.001	nw4	0.664	8.472	< 0.001
Zn, PI, NH3N, TP, DO	w5	0.780	6.699	< 0.001	nw5	0.815	5.681	< 0.001
Zn, PI, NH3N, TP, DO, Pb	w6	0.827	3.725	< 0.001	nw6	0.849	3.439	< 0.001
Zn, PI, NH3N, TP, DO, Pb, Cu	w7	0.915	3.146	< 0.001	nw7	0.926	2.642	< 0.001
Zn, PI, NH3N, TP, DO, Pb, Cu, COD	w8	0.955	3.820	< 0.001	nw8	0.958	3.302	< 0.001

**Figure 5:**
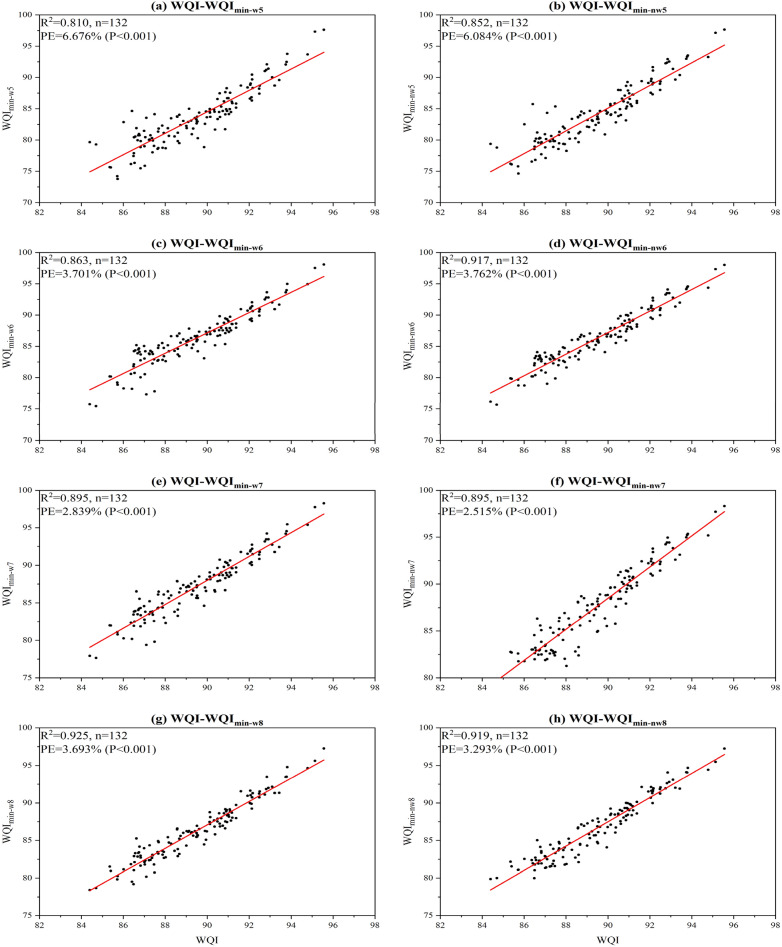
Comparison of the WQI and WQI_min_ values based on the testing dataset (the parameters for each WQI_min_ model are shown in Table 2).

Regarding the performance of WQI_min_ models in the test period (in Fig. 5), the WQI_min-nw7_ model still behaved well. It had an R^2^ value of 0.895 and a low PE of 5.515%. WQI_min-w5_/WQI_min-nw5_ model performed poorly with lower R^2^ yet higher PE; WQI_min-nw6_ model behaved well than WQI_min-w6_, WQI_min-w7_ or WQI_min-nw7_ models in terms of R^2^ but had a similar PE situation with WQI_min-nw5_; WQI_min-w8_ and WQI_min-nw8_ models showed slightly larger R^2^ values than WQI_min-nw7_ model whereas PE also increased a lot, nearly 46.84% (from 2.515% to 3.693%) and 30.93% (From 2.515% to 3.293%), respectively. Overall, WQImin-nw7 model could explain nearly 90% of the variations of WQI. The result demonstrated that WQI_min-nw7_ model had a powerful prediction ability, which was consistent with the training performance.

## Discussion

### Key parameter selection for WQI_min_ model

According to the stepwise multiple linear regression, the WQI_min_ model in this study consists of seven main parameters: Zn, PI, NH_3_^-^N, TP, DO, Pb and Cu. The WQI_min_ explains the variations of WQI well, and is more efficient for water quality assessment. Zn was the first parameter chosen for the regression, and it contributed most to the WQI evaluation in the training dataset (R^2^ > 0.40, *P* < 0.001). PI was the second significant sign for the variation of WQI, which represented the organic pollution of the water body. NH_3_^-^N and TP were the third and fourth parameters, respectively, on behalf of the nutritional parameters of water quality. Figure 2 also depicted that the Han River basin was threatened by a significant spatial variance of NH_3_^-^N and TP. In fact, previous literature has declared the impact of NH_3_^-^N and TP on water quality^25,47^. DO was the fifth parameter that could substantially improve R^2^. It is the main force of shaping the aquatic environment and biochemical process. It was also a crucial parameter appearing in the WQI_min_ model in other works. Pb and Cu were introduced into the model last, both reflected the heavy metals concentration and affected water quality. Heavy metals in the water body could also harm human health. More attention should be put on these two substances since they sometimes fail to reach the standard of Class I of water quality.

**Figure 6:**
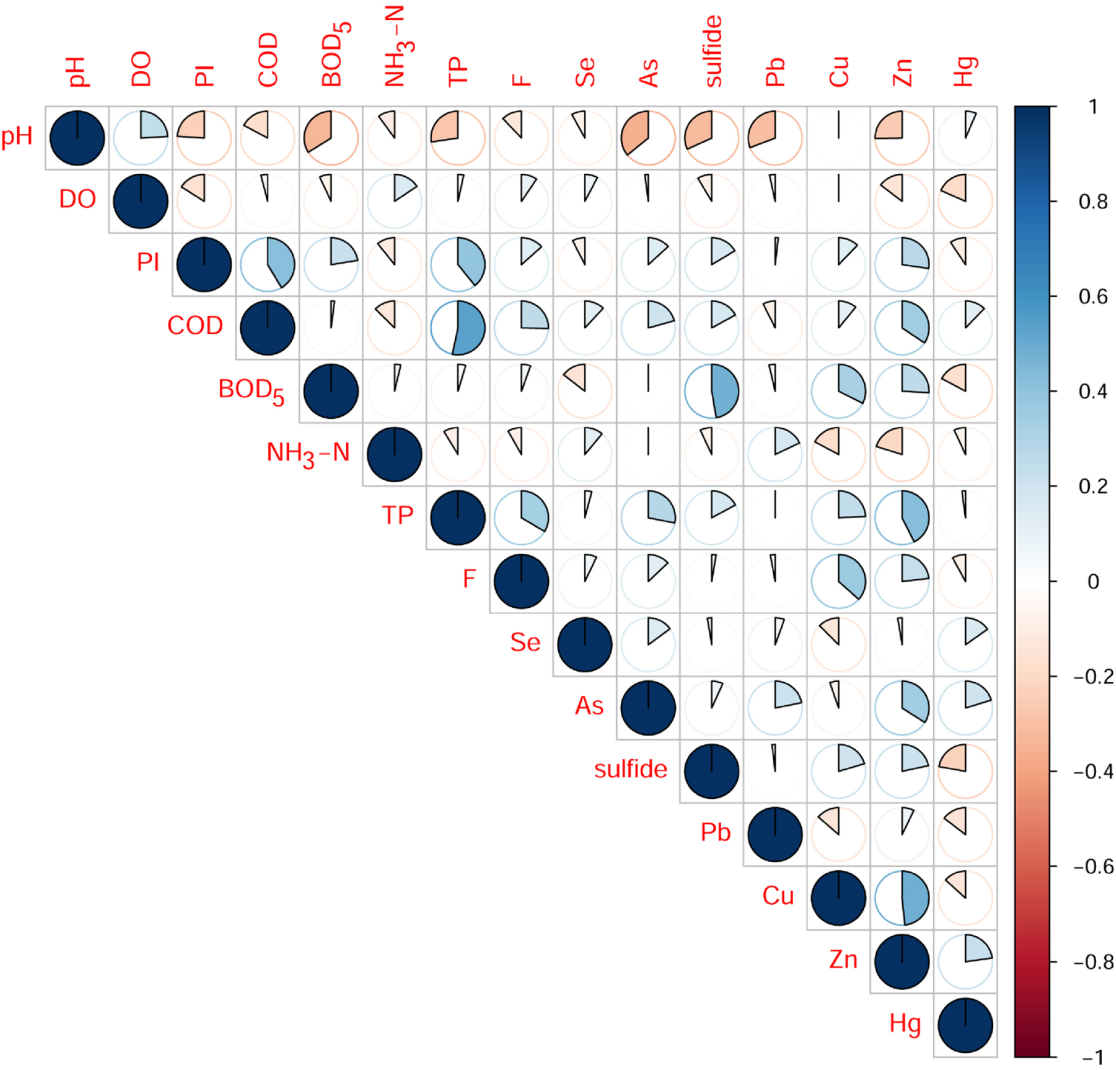
Correlation among water quality parameters during the period 2015 ~ 2017.

Except for the universal multiple linear regression, other methods can be employed and are found in some relevant studies. Hou et al.^48^ used principal component analysis (PCA) to identify the explanatory parameters for WQI variation. Additionally, a linear correlation analysis between parameters was also meaningful. It can be employed for reducing the number of parameters and selecting the key parameters for analysis. The Pearson’s linear correlation of physicochemical parameters was shown in Fig. 6. The colors signified the positive or negative correlations between parameters and the areas occupied by the clockwise rotation showed the strong or weak correlations. The results illustrated that pH had a strong correlation with many parameters except for Se, Cu, and Hg. DO is positively correlated with NH_3_^-^N but negatively with PI, Zn, and Hg. Positive correlations were identified between PI and COD, BOD_5_, TP, F-, As, sulfide, Cu, Zn. BOD_5_ exhibited positive correlations with sulfide, Cu and Zn but negative correlations with Se, and Hg. More elaborate results can be found in Fig. 6, which can make sense for the selection of key parameters.

### Impact sources and variation of water quality

Water quality evaluation can provide insights for the water resources exploitation and contamination control. In this study, we mainly utilized the measured data to unclose the water quality condition and developed a new method for evaluating the water quality. However, human activities, such as agricultural non-point source discharge, industrial and domestic sewage discharge, and anthropogenic intervention, could have a direct impact on the WQI performance. Some previous studies have found and reported this phenomenon. For example, Liu et al.^45^ found that farmland and urbanization could deteriorate water quality in the Han River basin. Tian et al.^49^ demonstrated that Lihu Lake suffer from the worst pollution during 1997–2003 due to excessive TN and TP. Although these parameters have improved significantly after artificial management, the last two years have witnessed a slight rebound. The overall evaluation revealed that the water quality could be taken as “good” or “excellent” levels in most cases and the upstream stations presented better water quality than downstream with some parameters like PI and NH_3_-N exceeded the thresholds, which implied that more effort can be made for the downstream water quality protection and the crucial water parameters needs to be constrained in these years. Moreover, the South-to-North Water Transfer Project and Yangtze-to-Han Water Transfer Project in recent decades also altered the hydrodynamic condition of the Han River to some extent and further affect water quality parameters. Han River has been confronted with emerging issues in these years and what exactly drives the change of the water quality might alter both in space and time still needs further investigation. For instance, how the operation of complex water transfer projects will impact the variation of the discharge and contamination concentration in middle and lower Han River is a hotspot issue^50^. Building a coupled hydrodynamic and water quality simulation model could contribute to the prediction of the contamination concentration and unveil the spatio-temporal variation. Furthermore, the simulation results that provide data sources under different hydrology or hydrodynamic condition can be coupled with the WQI and WQI_min_ model constructed in this study for achieving comprehensive water quality evaluation. The model coupled the physics-based hydrodynamic-water quality model and the statistical WQI model could shed light on the source of contamination and provide precise preventions and control measures in the changing environments. What exactly drives the change of the water quality in different sections and in different times still needs investigation. Due to data scarcity of wastewater discharge, urban expansion, and hydrodynamics, more results regarding impact factors remains explored to assist water quality management. The coupled model that can unveil the source and variation of the contamination and assess the comprehensive water quality under complex conditions is to be built in the further study.

### Uncertainty in water quality evaluation

Water quality evaluation is disturbed by multiple uncertainty sources, such as water sampling, measurement variability, water quality standard and water quality parameters. For example, both the weather and sampling time determine the DO content, and DO in aquatic ecosystems occupied the highest weight in water quality evaluation^25^. In this study, the water quality samplings were carried out on sunny or cloudy days in most cases to eliminate precipitation disturbance. Nevertheless, it was difficult to require all the samples following a strict schedule in such a huge study area. The inevitable laboratory measurement uncertainty might influence the confidence in WQI evaluations. Furthermore, the uncertainty was also induced by the water quality standards^17,51^. The classification standards vary in different districts and in different protection objectives and thus introduce some uncertainties in the assessment. In this study, the normalization values reconcile with the *Surface Water Quality Standards GB3838-2002*^39^, which conformed to the official guidance in China However, this standard system might not work for other regions or other protection objectives. The WQI evaluation results could be altered with the change of the standards. Another source of uncertainty is the assigned weights of water quality parameters^17^. In terms of the previous studies concerning the water quality evaluation, the weight allocated to each parameter showed large variation^52,53^. In some WQI aggregations, it is impractical to use a unit weight for each parameter due to their different levels of impact on the water status. The high concentration of a parameter with high weight could translate to a low WQI value resulting in a misunderstanding of the water quality condition. Therefore, a proper approach for assigning the weights is essential. The weights used in this study were adopted and revised from Pesce et al.^19^ and Sun et al.^54^, which has also been verified in other studies. Whereas the uncertainty of WQI weights has not been investigated in this study, which is out of the main scope of the research. To further consider the overall uncertainty in WQI evaluation in the next steps, the statistical uncertainties with respect to the water quality parameters can be investigated using Monte-Carlo simulation^17^.

## Conclusions

The water quality condition of the middle and lower Han River basin in China from 2015 to 2017 and the spatio-temporal variation of 15 water quality parameters were investigated and analyzed in this study. The water quality from seasonal and spatial scales was comprehensively evaluated with the WQI method and the key parameters were selected to develop the WQI_min_ model. The main conclusions are as follows:

1. Eight parameters, i.e., PI, COD, TP, F^-^, As, Pb, Cu and Zn, performed obvious spatial discrepancy. The physicochemical and nutrient parameters, except for PI and NH_3_^-^N, satisfied the standard of Class I during the period 2015–2017. The heavy metal parameters in the middle and lower reaches of the Han River basin stayed at a low level, while Cu, Zn, and Pb exceeded the threshold.
2. The water qualities of this study basin were evaluated as “good” and “excellent” in most cases, with the mean WQI values of stations and seasons varying from 87.07 to 92.33 and from 87.43 to 90.33, respectively. On a spatial scale, the upstream stations presented better water quality than downstream; on a temporal scale, the autumn season was found the appearance time of worst water quality in 2015 and 2017 and the summer season was replaced in 2016.
3. The WQI_min_ model developed in this study consisted of seven key parameters, i.e., Zn, PI, NH_3_^-^N, TP, DO, Pb and Cu. It has more physical explanatory and better evaluation performance than WQI for water quality evaluation.

Given the study area to be the strategic water source of China and the core of the Han River ecological economic belt, more efforts on linkage between water quality and United Nations-Water Sustainable Development Goal 6 are necessary and recommended in the future.

## Supplementary Information

Supplementary Information.

## Data Availability

The datasets used and/or analysed during the current study available from the corresponding authors on reasonable request.

## Code availability

The code applications used in this work can be obtained by contacting the corresponding authors.
