# Supplementary material for: Spatiotemporal variation evaluation of water quality in middle and lower Han River, China
Source: Sci Rep. 2022 Aug 19;12:14125. doi: 10.1038/s41598-022-16808-w (PMC9391420; doi:10.1038/s41598-022-16808-w)
Supplement: Supplementary file 1 — Supplementary Information. [file 41598_2022_16808_MOESM1_ESM.docx]

**Table S1**

The location of the water quality monitoring stations in middle and lower reaches of Han River basin in China.

| **Stations** | **Abbreviation** | **Section code** | **City/County** |
| --- | --- | --- | --- |
| Shenwan | SW | 402 | Xiangyang City |
| Baijiawan | BJW | 1 | Xiangyang City |
| Yujiahu | YJH | 251 | Xiangyang City |
| Zhuandou | ZD | 404 | Zhongxiang City |
| Huangzhuang | HZ | 411 | Zhongxiang City |
| Luohanzha | LHZ | 406 | Jingmen City |
| Zekou | ZK | 511 | Qianjiang City |
| Shidou | SD | 408 | Xiantao City |
| Hannancun | HNC | 505 | Xiantao City |
| Xingou | XG | 410 | Wuhan City |
| Zongguan | ZG | 51 | Wuhan City |

**Table S2**

The normalization values and weights of water quality parameters used in the WQI calculation based on the *Environmental Quality Standards for Surface Water of China* (GB 3838-2002) in this study.

| **Parameters** | **Units** | **Weight^a^** | **Surface Water Environmental Quality Standards** | | | | |
| --- | --- | --- | --- | --- | --- | --- | --- |
|  |  | **(*P_i_*)** | **I** | II | **III** | **IV** | **V** |
|  |  |  | $\boldsymbol{I}_{\boldsymbol{i}\mathbf{,1}}$**= 20^b^** | $\boldsymbol{I}_{\boldsymbol{i}\mathbf{,2}}$**= 40^b^** | $\boldsymbol{I}_{\boldsymbol{i}\mathbf{,3}}$**= 60^b^** | $\boldsymbol{I}_{\boldsymbol{i}\mathbf{,4}}$**= 80^b^** | $\boldsymbol{I}_{\boldsymbol{i}\mathbf{,5}}$**= 100^b^** |
| pH **^c^** | - | 1 | 6~9 | | | | |
| DO | mg/L | 4 | 7.5 | 6 | 5 | 3 | 2 |
| PI | mg/L | 3 | 2 | 4 | 6 | 10 | 15 |
| COD | mg/L | 3 | 15 | 15 | 20 | 30 | 40 |
| BOD_5_ | mg/L | 3 | 3 | 3 | 4 | 6 | 10 |
| NH_3_^-^N | mg/L | 3 | 0.15 | 0.5 | 1 | 1.5 | 2 |
| TP | mg/L | 4 | 0.02 | 0.1 | 0.2 | 0.3 | 0.4 |
| F^−^ | mg/L | 2 | 1 | 1 | 1 | 1.5 | 1.5 |
| Se | μg/L | 4 | 10 | 10 | 10 | 20 | 20 |
| As | μg/L | 4 | 50 | 50 | 50 | 100 | 100 |
| Sulfide | mg/L | 2 | 0.05 | 0.1 | 0.2 | 0.5 | 1.0 |
| Pb | μg/L | 4 | 0.01 | 0.01 | 0.05 | 0.05 | 0.1 |
| Cu | μg/L | 2 | 10 | 1000 | 1000 | 1000 | 1000 |
| Zn | μg/L | 2 | 50 | 1000 | 1000 | 2000 | 2000 |
| Hg | μg/L | 4 | 0.05 | 0.05 | 0.1 | 1 | 1 |

^a^ The weights are adopted and revised from [1, 2, 3].

^b^ The normalization values are according to the *Surface Water Quality Standards GB*3838-2002 [4].

^c^ The calculation of pH is adopted from [5].

**References**

1. Koçer MAT, Sevgili H (2014) Parameters selection for water quality index in the assessment of the environmental impacts of land-based trout farms. Ecol Indic 36:672–681. https://doi.org/10.1016/j.ecolind.2013.09.034
2. Pesce SF, Wunderlin DA (2000) Use of water quality indices to verify the impact of Córdoba City (Argentina) on Suquı́a River. Water Res 34:2915–2926. https://doi.org/10.1016/S0043-1354(00)00036-1
3. Sun W, Xia C, Xu M, et al (2016) Application of modified water quality indices as indicators to assess the spatial and temporal trends of water quality in the Dongjiang River. Ecol Indic 66:306–312. https://doi.org/10.1016/j.ecolind.2016.01.05
4. Ministry of Environmental Protection, China (2002) Environmental Quality Standards for Surface Water (GB3838-2002). https://english.mee.gov.cn/Resources/standards/water_environment/quality_standard/200710/t20071024_111792.shtml
5. Hou W, Sun S, Wang M, et al (2016) Assessing water quality of five typical reservoirs in lower reaches of Yellow River, China: Using a water quality index method. Ecol Indic 61:309–316. https://doi.org/10.1016/j.ecolind.2015.09.030

**Table S3**

## Linear models explaining the WQI (lg(WQI+1)) based on the training data set. The models result from a stepwise selection procedure using all 15 parameters examined in our study after normalization.

| Model | Linear model | R^2^ | *P* |
| --- | --- | --- | --- |
| 1 | 1.548^***^+0.205^***^lg(Zn+1) | 0.411 | <0.001 |
| 2 | 1.386^***^+0.180^***^lg(Zn +1)+0.111^***^lg(PI+1) | 0.582 | <0.001 |
| 3 | 1.225^***^+0.202^***^lg(Zn+1)+0.107^***^lg(PI+1)+0.066lg^***^(NH_3_-N +1) | 0.690 | <0.001 |
| 4 | 1.151^***^+0.182^***^lg(Zn+1)+0.096^***^lg(PI+1)+0.066^***^lg(NH_3_-N+1)+0.073^***^lg(TP+1) | 0.753 | <0.001 |
| 5 | 1.020^***^+0.147^***^lg(Zn+1)+0.083^***^lg(PI+1)+0.070^***^lg(NH_3_-N+1)+0.101^***^lg(TP+1)+0.083^***^lg(DO) | 0.857 | <0.001 |
| 6 | 0.942^***^+0.122^***^lg(Zn+1)+0.087^***^lg(PI+1)+0.063^***^lg(NH_3_-N+1)+0.102^***^lg(TP+1)+0.087^**^lg(DO)+0.063^***^lg(Pb+1) | 0.891 | <0.001 |
| 7 | 0.865^***^+0.078^***^lg(Zn+1)+0.081^***^lg(PI+1)+0.067^***^lg(NH_3_-N+1)+0.089^***^lg(TP+1)+0.088^***^lg(DO+1)+0.090^***^lg(Pb+1)+0.070^***^lg(Cu+1) | 0.936 | <0.001 |
| 8 | 0.807^***^+0.077^***^lg(Zn+1)+0.073^***^lg(PI+1)+0.066^***^lg(NH_3_-N+1)+0.075^***^lg(TP)+0.089^**^lg(DO+1)+0.090^***^lg(Pb+1)+0.065^***^lg(Cu+1)+0.057^***^lg(COD+1) | 0.964 | <0.001 |

n=264. ^***^*P*<0.001.


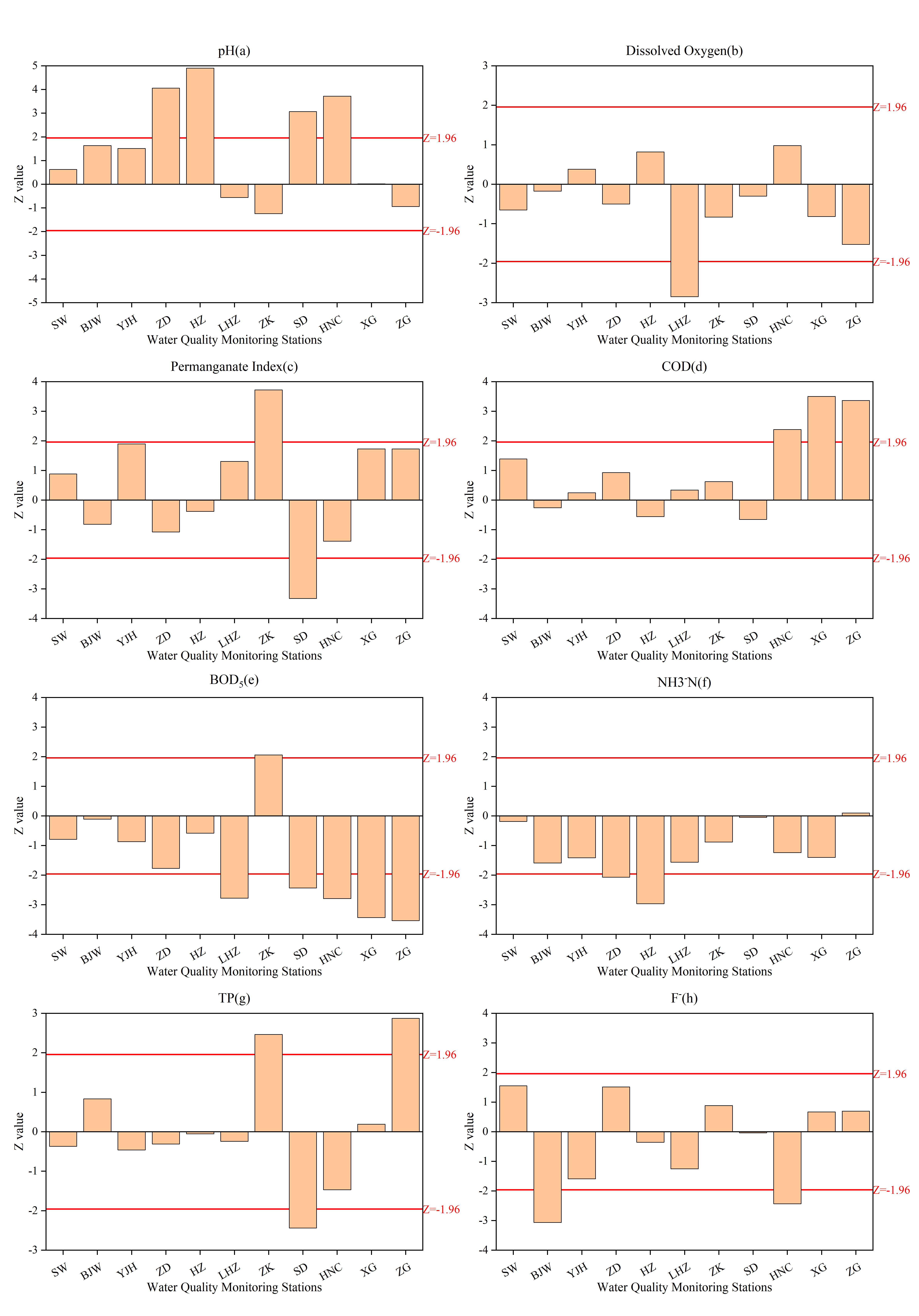


**Fig. S1.** The results of Mann-Kendall test for 15 water quality parameters for each water quality monitoring station in middle and lower reaches of Han River basin in China from 2015 to 2017.


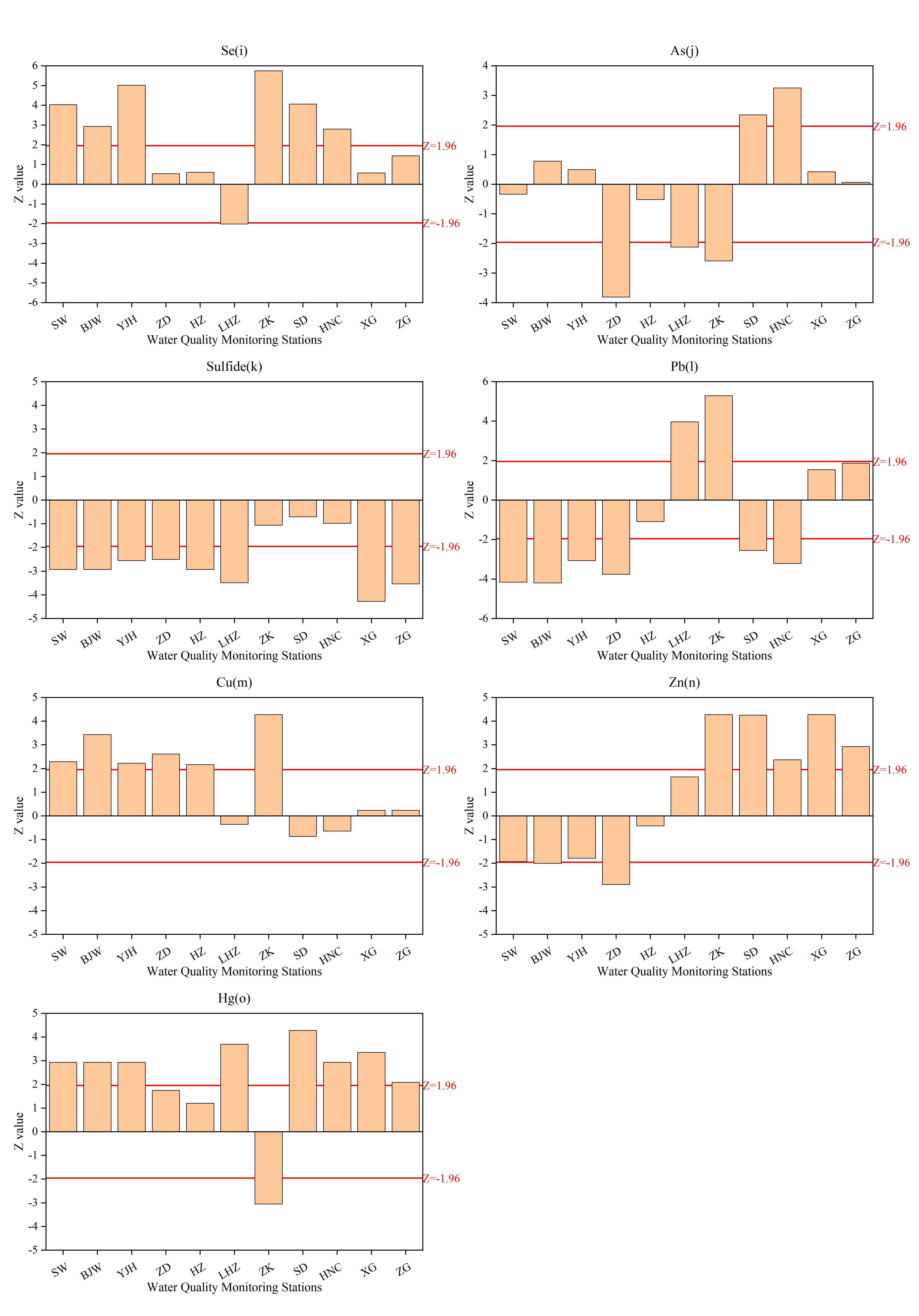


**Fig. S1.** (continued).
